# Trajectories of triglyceride-glucose index changes and their association with all-cause and cardiovascular mortality: a competing risk analysis

**DOI:** 10.1186/s12933-024-02457-y

**Published:** 2024-10-15

**Authors:** Jun-Hyuk Lee, Soyoung Jeon, Hye Sun Lee, Ji-Won Lee

**Affiliations:** 1 Department of Family Medicine, Nowon Eulji Medical Center, Eulji University School of Medicine, Seoul, 01830 Republic of Korea; 2 Department of Research Affairs, Biostatistics Collaboration Unit, Yonsei University College of Medicine, Seoul, 03277 Republic of Korea; 3 Department of Family Medicine, Yonsei University College of Medicine, 50-1 Yonsei-ro, Seodaemun-gu, Seoul, 03722 Republic of Korea; 4 Institute for Innovation in Digital Healthcare, Yonsei University, Seoul, 03722 Republic of Korea

**Keywords:** TyG index, Insulin resistance, Trajectory, Cardiovascular disease, Mortality

## Abstract

**Background:**

The association between changes in insulin resistance, reflected by the triglyceride-glucose (TyG) index, and mortality remains unclear. This study investigated whether longitudinal trajectories of TyG index changes are associated with all-cause and cardiovascular disease (CVD) mortality.

**Methods:**

This retrospective cohort study analyzed data from 233,546 adults aged ≥ 19 years from the Korea National Health Insurance Service-National Sample Cohort. Participants were categorized as having increasing, stable, or decreasing TyG index changes during a 4-year exposure period (2009–2014). Mortality outcomes were assessed during an 8.13-year follow-up period (2015–2021). Cox proportional hazards regression and competing risk analysis were used to evaluate all-cause and CVD mortality.

**Results:**

A total of 7918 mortality events, including 651 CVD deaths, were recorded. Compared with the stable group, adjusted hazard ratios for all-cause mortality were 1.09 (95% CI 1.03–1.15) in the increasing group and 1.23 (95% CI 1.01–1.50) for CVD mortality. An increased TyG index was significantly associated with all-cause mortality in individuals aged < 50 years; men; and individuals with obesity, hypertension, diabetes, and/or dyslipidemia. For CVD mortality, significant associations were found in individuals aged 50–69 years, with obesity, with diabetes, or without dyslipidemia.

**Conclusion:**

An increasing TyG index from baseline during follow-up was independently associated with higher risks of all-cause and CVD mortality. Serial monitoring of TyG index changes could enhance risk stratification and inform targeted interventions to reduce insulin resistance, and ultimately lower mortality risk.

**Supplementary Information:**

The online version contains supplementary material available at 10.1186/s12933-024-02457-y.

## Introduction

Cardiovascular disease (CVD) remains the leading cause of mortality globally, accounting for 34.9% of all deaths in 2022 [1]. In 2021, ischemic heart disease had an age-standardized mortality rate of 108.7, making it the leading cause of death globally, whereas stroke ranked third with a mortality rate of 87.4 [2]. Insulin resistance (IR) is a crucial driver of atherosclerosis and cardiovascular disease (CVD) progression [3, 4]. The hyperinsulinemic–euglycemic clamp is the gold standard for measuring IR, but its complexity limits its clinical use. The triglyceride-glucose (TyG) index has recently gained attention as a simpler, noninvasive surrogate marker for IR [5]. Studies suggest that the TyG index may outperform homeostatic model assessment for IR (HOMA-IR) in predicting cardiovascular and metabolic outcomes because it reflects lipid and glucose metabolism, thereby providing a more comprehensive view of metabolic health [6–9].

To date, most studies have relied on single-point measurements of IR, which fail to capture dynamic changes over time. Similar to how blood pressure changes occur over a lifespan [10, 11], IR can also increase, remain stable, or decrease over time. Therefore, tracking changing trends of IR could reflect CVD risk, thereby improving risk stratification and potentially more targeted interventions. Some studies indicate that increasing HOMA-IR trajectories are correlated with higher CVD risk and mortality [12], and other studies have explored TyG index changes by using two timepoints [13]. However, these approaches may overlook significant fluctuations in IR over time. The relationship between long-term TyG index trajectories and CVD outcomes, particularly the impact of decreasing values, remains underexplored.

In this context, we hypothesized that long-term trajectories of TyG index changes are associated with CVD outcomes in the Korean population. By using a large Korean cohort, this study conducted a comprehensive longitudinal analysis to explore how dynamic shifts in TyG index trajectories (i.e., increase, decrease, stable) influence long-term CVD and mortality outcomes.

## Methods

### Study design and population

This retrospective cohort study utilized data from the Korea National Health Insurance Service-National Sample Cohort (NHIS-NSC) 2.2 database. The Korean National Health Screening program conducts biennial health screenings. In 2009, the program began measuring serum triglyceride levels, which is essential for calculating the TyG index. Baseline data from the 2009–2010 NHIS-NSC comprised information on 363,270 participants, with follow-up data available until December 31, 2021. To identify TyG index trajectories, we required data from at least three timepoints while maximizing the event accrual period. Therefore, we defined 2009–2014 as the exposure period, during which TyG index changing patterns were assessed, and 2015–2021 as the event accrual period, during which mortality outcomes were tracked. Mortality status was determined by linking personal identification key codes generated by the NHIS-NSC with national data sources, including death records from the Korea National Statistical Office.

**Fig. 1:**
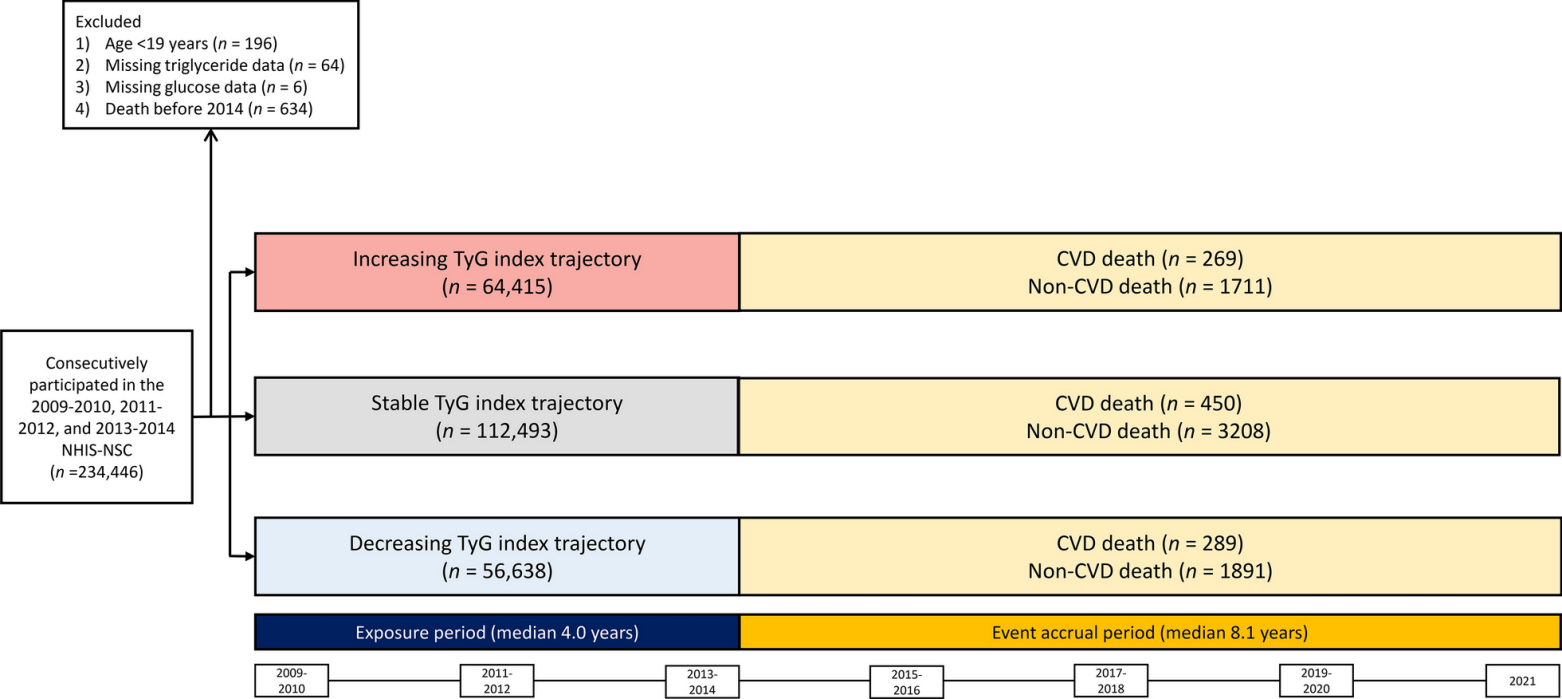
Flowchart of the study population. Abbreviations: TyG, triglyceride-glucose; CVD, cardiovascular disease; NHIS-NSC, National health insurance service-national sample cohort

Figure 1 illustrates the flowchart of the study population. Among the 363,270 participants in the 2009–2010 NHIS-NSC, we selected 234,446 individuals who consecutively participated in the NHIS-NSC during the 2009–2010, 2011–2012, and 2013–2014 periods. We excluded (1) individuals aged < 19 years (n = 196), (2) individuals with missing serum triglyceride data (n = 64), (3) individuals with missing glucose data (n = 6), and (4) individuals who died before 2014 (n = 634). Finally, 233,546 participants were included in the analysis.

This study was conducted in accordance with the STROBE Statement. The Institutional Review Board of Eulji University Hospital (Seoul, Republic of Korea) approved the study (approval no. 2023-12-018). The requirement for informed consent was waived because we used anonymized data provided by the NHIS-NSC database, based on the Personal Data Protection Act guidelines.

### Trajectories of TyG index changes

**Fig. 2:**
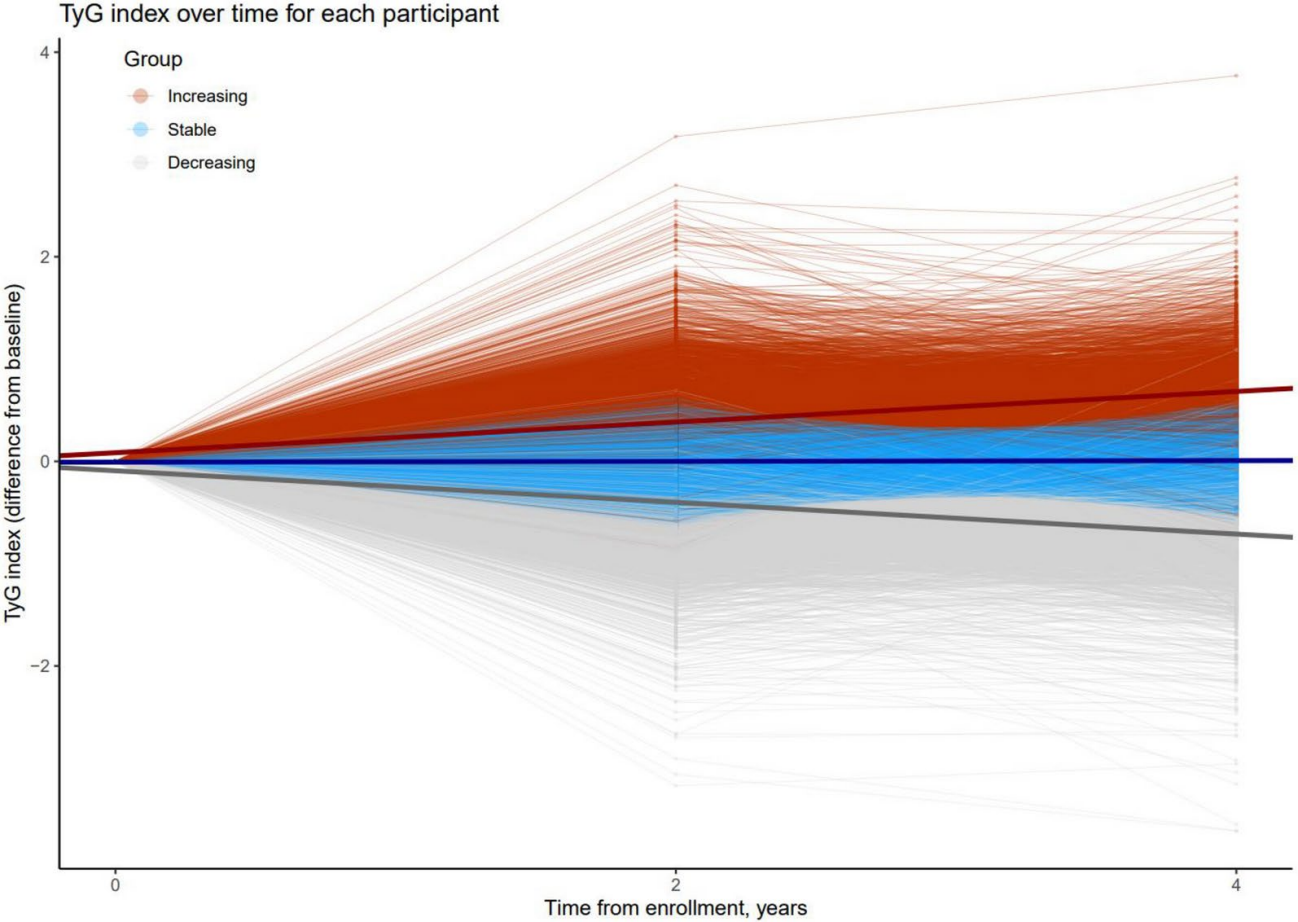
Changes in the TyG index of each individual during the exposure period. Abbreviations: TyG, triglyceride-glucose

The TyG index was calculated, using the following formula [5]: TyG index = ln [triglyceride (mg/dL) × glucose (mg/dL) ÷ 2]. We classified participants into the increasing (*n* = 64,415), stable (*n* = 112,493), and decreasing (*n* = 56,638) TyG index trajectory groups from baseline through follow-up by using Gaussian finite mixture modeling (Supplementary Table 1 and Fig. 2). Detailed information on the method for trajectory modeling is described in the statistical analysis section.

### Outcomes

The primary outcome was all-cause mortality. Mortality status information was linked to the Korea National Statistical Office, which recorded death codes, based on the International Classification of Diseases, Tenth Revision (ICD-10). The secondary outcome was CVD mortality, which included ischemic heart disease (ICD-10 codes I20–I25) and cerebrovascular disease (ICD-10 codes I60–I69). All mortality events between January 2015 and December 2021 were recorded.

### Covariates

Height and body weight were measured to the nearest 0.001 m and 0.1 kg, respectively. The body mass index (BMI) was calculated by dividing body weight by height squared (kg/m^2^), with obesity defined as a BMI ≥ 25 kg/m^2^ [14]. After at least 5 min of resting, the systolic blood pressure (SBP) and diastolic blood pressure (DBP) were measured in the seated position. The levels of fasting blood glucose (FBG), serum creatinine, total cholesterol, triglyceride, and high-density lipoprotein cholesterol (HDL-C) were measured. The estimated glomerular filtration rate (eGFR) was calculated using the Modification of Diet in Renal Disease Study equation [15]. Participants were classified as current smokers or nonsmokers, based on their smoking status, and as current drinkers or nondrinkers, based on their alcohol consumption status. Regular exercise was defined as moderate physical activity ≥ 150 min/week or vigorous physical activity ≥ 75 min/week. A diagnosis of diabetes mellitus (DM) was based on the presence of at least one of the following criteria: (1) FBG ≥ 126 mg/dL, (2) current treatment with DM medication, or (3) ICD-10 codes E11–E14. Participants were considered to have hypertension (HTN) if they met at least one of the following criteria: (1) SBP ≥ 140 mmHg, (2) DBP ≥ 90 mmHg, (3) current treatment with HTN medication, or (4) ICD-10 codes I10–I13. Dyslipidemia (DLD) was defined as the presence of at least one of the following criteria: (1) serum total cholesterol ≥ 240 mg/dL, (2) current treatment with DLD medication, or (3) ICD-10 code E78.

### Statistical analysis

Gaussian finite mixture modeling was performed to identify different trajectories of changes in the TyG index from baseline TyG index values during the median 4.0-year exposure period by using the command ‘lcMethodMclust’ in R package ‘latrend’ [16]. This method employs the expectation–maximization (EM) algorithm to estimate the parameters of the mixture model iteratively. The EM algorithm alternates between assigning individuals to latent trajectory groups (i.e., expectation step) and updating group-specific parameters to maximize the likelihood of the observed data (i.e., maximization step). This approach allows identifying underlying patterns and heterogeneity in the longitudinal changes of the TyG index, thereby facilitating a clearer understanding of its variations across different groups within the study population. The criteria for selecting the optimal number of groups were (1) a low Bayesian information criterion, (2) minimum group size exceeding 20% of the total sample, and (3) at least one group with an average posterior probability assignment of 90% or higher.

All data are presented as the mean ± standard deviation or as the median (interquartile range [IQR]: 25th and 75th percentiles) for continuous variables and as the number (percentage [%]) for categorical variables. For group comparisons, analysis of variance was used for continuous variables that satisfied the normality assumption, whereas the Kruskal–Wallis test was used for variables that did not. The chi-square test was applied to categorical variables. An adjusted survival curve was drawn, depicting the survival probability, adjusted for the TyG index at baseline. Additionally, we created an adjusted survival curve by using a proportional subdistribution hazard model to illustrate the cumulative incidence rates of CVD mortality, based on the TyG index trajectory groups, which were also adjusted for the TyG index at baseline [17]. For the primary outcome, Cox proportional hazards regression analysis was conducted to estimate the hazard ratio (HR) with a 95% confidence interval (CI) for the all-cause mortality of the increasing and decreasing TyG index trajectory groups, compared with that of the stable group. The Cox proportional hazards assumption using the Schoenfeld residuals test confirmed that the assumption was met (*P* = 0.71).

For the secondary outcome, competing risk analysis was conducted by using the Fine and Gray model to estimate the subdistribution HR with a 95% CI for CVD death, setting non-CVD mortality as a competing risk. The TyG index at baseline was adjusted in Model 1. Age, sex, BMI, and the TyG index at baseline were adjusted in Model 2. In Model 3, smoking status, drinking status, and regular exercise were adjusted, in addition to the variables used in Model 2. In Model 4, eGFR, HTN, DM, and DLD were adjusted, in addition to the variables used in Model 3. Subgroup analyses were conducted, based on age group (< 50 years, 50–69 years, ≥ 70 years), sex, obesity, HTN, DM, and DLD status. Sensitivity analysis was conducted by using an alternative trajectory modeling approach via group-based trajectory modeling with fixed-effects. We also performed another sensitivity analysis by repeating the analysis starting from trajectory modeling, after excluding 765 individuals who died during the first 2 years of the event accrual period.

All statistical analyses were conducted using R (version 4.3.1; R Foundation for Statistical Computing, Vienna, Austria) and SAS statistical software (version 9.4; SAS Institute Inc., Cary, NC, USA). A two-sided *P* value of < 0.05 was statistically significant.

## Results

### Clinical characteristics of the study population

Table 1 shows the baseline characteristics of the study population. The mean age of the 233,546 participants was 47.9 ± 13.3 years, and 53.6% were men. The decreasing group had the highest mean age, BMI, SBP, DBP, FBG and serum total cholesterol levels, along with the highest median serum triglyceride level, lowest serum HDL-C and eGFR, and the highest proportion of patients with HTN, DM, and DLD. The increasing group had the highest proportion of current smokers, current drinkers, and regular exercisers.

**Table 1:** Clinical characteristics of the study population based on the TyG index trajectories

	TyG index trajectory groups			
	Increasing	Stable	Decreasing	*P* value
	(n = 64,415)	(n = 112,493)	(n = 56,638)	
Men, n (%)	35,385 (54.9%)	58,225 (51.8%)	31,641 (55.9%)	< 0.001
Age, years	46.6 ± 13.3	47.9 ± 13.4	49.3 ± 13.1	< 0.001
BMI, kg/m^2^	23.7 ± 3.1	23.7 ± 3.1	24.1 ± 3.1	< 0.001
SBP, mmHg	121.6 ± 14.5	121.9 ± 14.7	124.2 ± 15.0	< 0.001
DBP, mmHg	75.8 ± 9.9	76.0 ± 9.9	77.4 ± 10.0	< 0.001
FBG, mg/dL	93.1 ± 17.9	95.7 ± 17.7	104.9 ± 32.0	< 0.001
Total cholesterol, mg/dL	191.7 ± 38.9	195.5 ± 39.6	201.6 ± 44.1	< 0.001
Triglyceride, mg/dL	82 (58, 118)	106 (76, 151)	160 (113, 234)	< 0.001
HDL-C, mg/dL	55 (46, 64)	54 (45, 63)	51 (43, 61)	< 0.001
Creatinine, mg/dL	0.9 (0.8, 1.0)	0.9 (0.8, 1.0)	0.9 (0.8, 1.1)	< 0.001
eGFR, mL/min/1.73m^2^	79.9 (70.3, 91.8)	79.1 (69.4, 90.9)	78.0 (68.3, 90.5)	< 0.001
Current smoker, n (%)	16,705 (26.1%)	24,720 (22.1%)	12,934 (22.9%)	< 0.001
Current drinker, n (%)	31,152 (48.4%)	50,724 (45.1%)	26,433 (46.7%)	< 0.001
Regular exerciser, n (%)	16,945 (26.6%)	28,023 (25.2%)	14,224 (25.4%)	< 0.001
HTN, n (%)	23,428 (36.4%)	41,761 (37.1%)	24,814 (43.8%)	< 0.001
DM, n (%)	4416 (6.9%)	7068 (6.3%)	7784 (13.7%)	< 0.001
DLD, n (%)	7424 (11.5%)	14,476 (12.9%)	9590 (16.9%)	< 0.001
TyG index				
2009–2010	8.3 ± 0.6	8.5 ± 0.6	9.0 ± 0.6	< 0.001
2011–2012	8.8 ± 0.7	8.5 ± 0.6	8.5 ± 0.7	< 0.001
2013–2014	8.9 ± 0.7	8.6 ± 0.6	8.4 ± 0.6	< 0.001

*TyG* triglyceride-glucose, *BMI* body mass index, *SBP* systolic blood pressure, *DBP* diastolic blood pressure, *FBG* fasting blood glucose, *HDL-C* high-density lipoprotein cholesterol, *eGFR* estimated glomerular infiltration rate, *HTN* hypertension, *DM* diabetes mellitus, *DLD* dyslipidemia

### Risk of all-cause mortality, based on the TyG index change trajectory

**Fig. 3:**
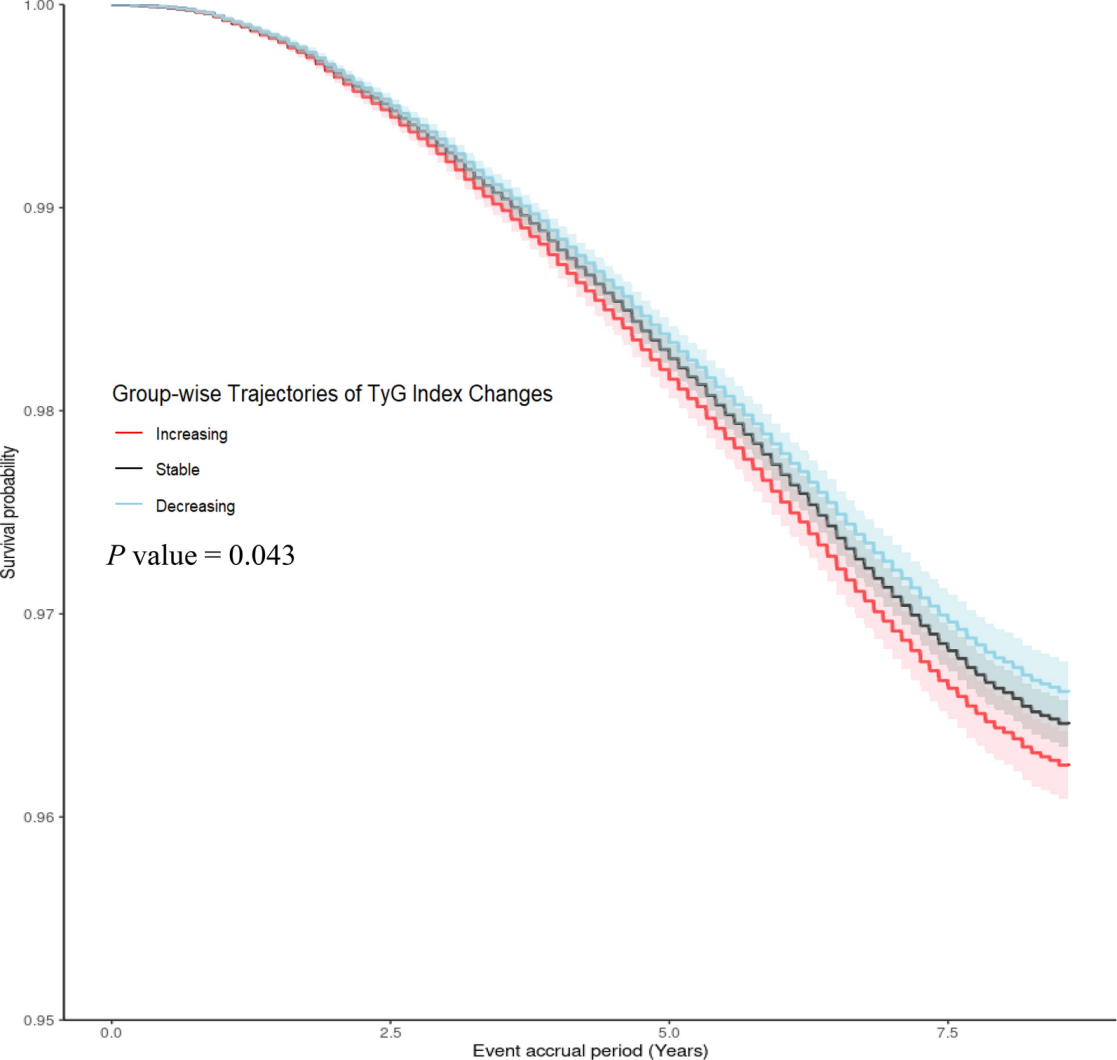
Adjusted survival plot showing survival probability according to the TyG index trajectories, adjusted for the TyG index at baseline. Abbreviations: TyG, triglyceride-glucose

During the median 8.13-year event accrual period, 7918 death events occurred. Figure 3 depicts the survival probability curves, which were adjusted for the TyG index at baseline, based on the TyG index trajectory group during the event accrual period. Survival probability was the lowest in the increasing group, followed by the stable and decreasing groups, respectively (*P* = 0.043).

Table 2 presents the results of the Cox proportional hazards regression analysis. In the multivariable models, the increasing group exhibited a significantly higher risk of all-cause mortality than that in the stable group. The adjusted HR (95% CI) for all-cause mortality in the increasing group was 1.06 (1.00–1.12) in Model 1, 1.17 (1.11–1.24) in Model 2, 1.14 (1.08–1.21) in Model 3, and 1.09 (1.03–1.15) in Model 4. The multivariable models did not reveal significant differences in mortality risk between the stable and decreasing groups.

**Table 2:** Cox proportional hazard regression analysis for all-cause mortality by trajectory groups of TyG index changes

	TyG index trajectory groups				
	Increasing (n = 64,415)		Stable (n = 112,493)	Decreasing (n = 56,638)	
	HR (95% CI)	*P* value	HR	HR (95% CI)	*P* value
All-cause moratlity					
Model 1	1.06 (1.00–1.12)	0.043	1 (ref)	0.95 (0.90–1.01)	0.108
Model 2	1.17 (1.11–1.24)	< 0.001	1 (ref)	0.96 (0.91–1.02)	0.172
Model 3	1.14 (1.08–1.21)	< 0.001	1 (ref)	0.97 (0.91–1.03)	0.266
Model 4	1.09 (1.03–1.15)	0.003	1 (ref)	0.97 (0.91–1.03)	0.266

Model 1: adjusted for TyG index at baseline

Model 2: adjusted for TyG index at baseline, age, sex, and BMI

Model 3: adjusted for variables used in Model 2 plus smoking status, drinking status, and regular exercise

Model 4: adjusted for variables used in Model 3 plus eGFR, HTN, DM, and DLD

*TyG* triglyceride-glucose, *BMI* body mass index, *eGFR* estimated glomerular filtration rate, *HTN* hypertension, *DM* diabetes mellitus, *DLD* dyslipidemia, *HR* hazard ratio, *CI* confidence interval

Supplementary Table 2 presents the results of the subgroup analysis for all-cause mortality. The fully adjusted model revealed that the increasing TyG index trajectory group had a significantly higher risk of all-cause death in the subgroups of individuals aged < 50 years, men, obesity, HTN, DM, and DLD.

### Risk of CVD mortality, based on the TyG index change trajectory

**Fig. 4:**
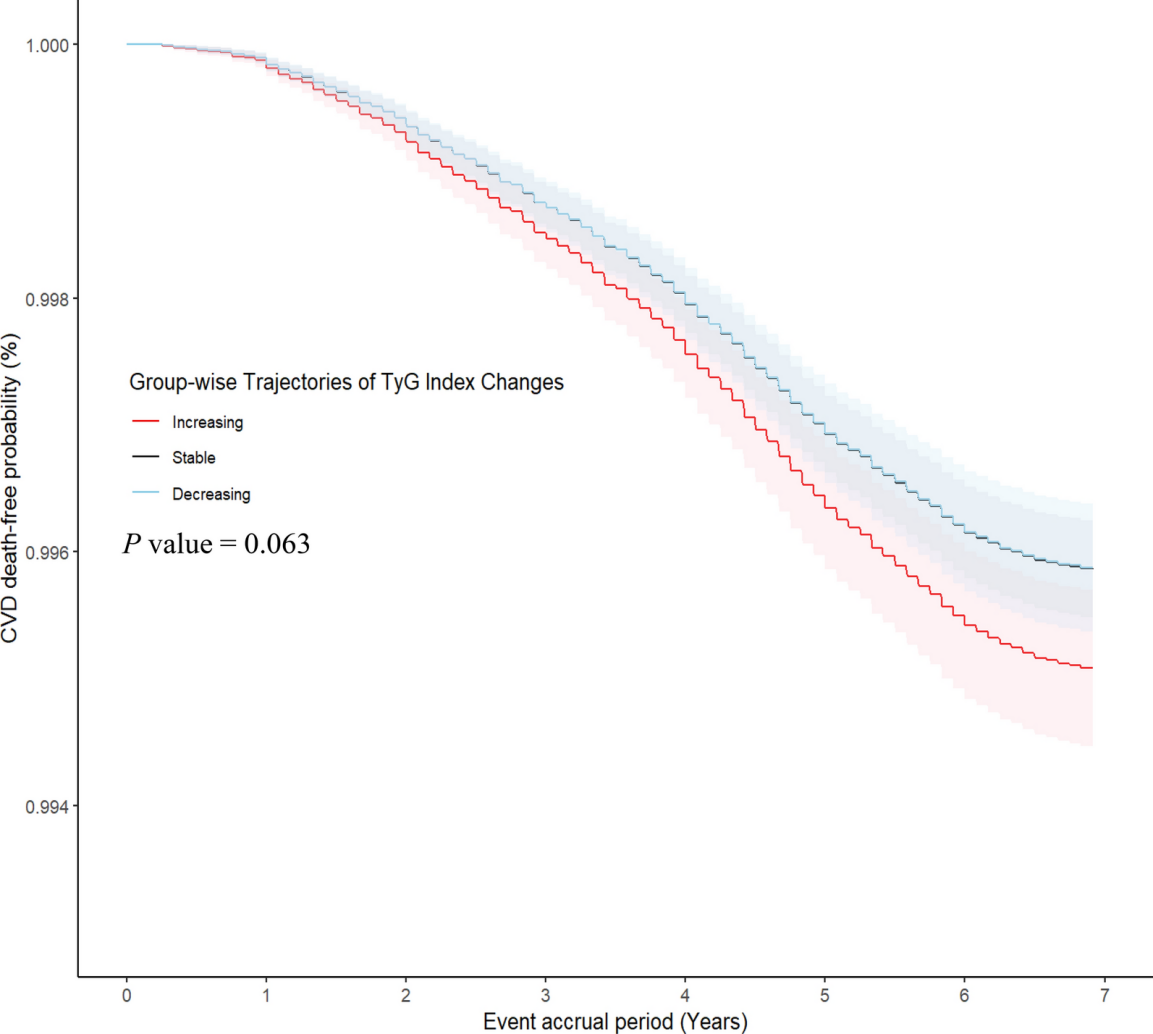
Adjusted survival plot showing cumulative CVD death-free probability according to the TyG index trajectories, adjusted for the TyG index at baseline. Abbreviations: TyG, triglyceride-glucose; CVD, cardiovascular disease

**Fig. 5:**
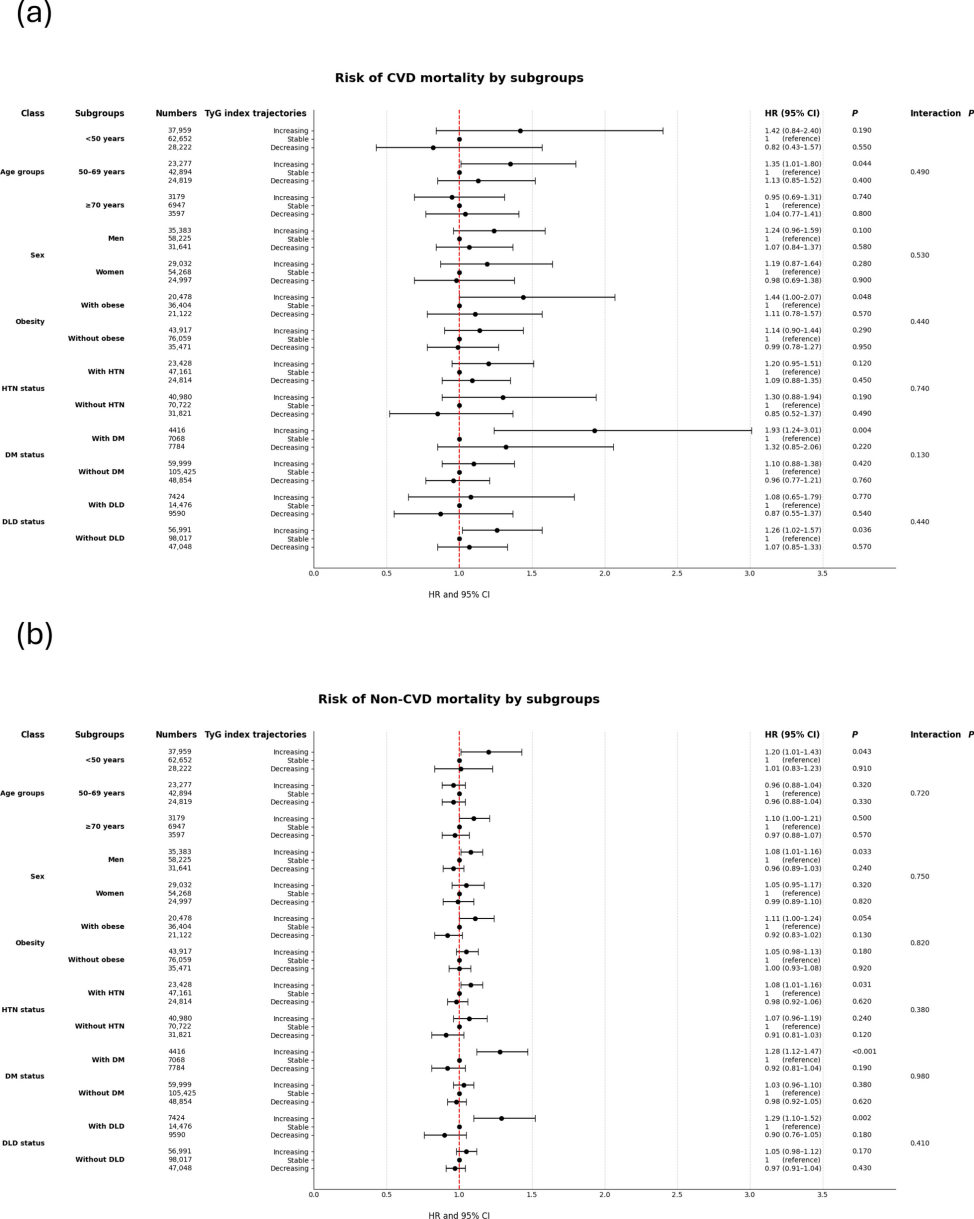
Forest plots of the results of the subgroup analysis for CVD mortality (**a**), considering non-CVD mortality (**b**) as a competing risk. Abbreviation: CVD, cardiovascular disease

A total of 651 CVD deaths and 7267 non-CVD deaths occurred during the event accrual period. The cumulative incidence rates of CVD death, adjusted for the TyG index at baseline, are depicted, based on the three TyG index trajectory groups, in Fig. 4. The increasing group exhibited a higher cumulative CVD death rate than that of the other groups, with a trend toward significance (*P* = 0.063). Table 3 presents the risk of CVD mortality, based on the TyG index trajectories, using competing risk analysis. In the multivariable models, the adjusted HR (95% CI) for CVD death in the increasing group, compared with the stable group, was 1.20 (0.99–1.46) in Model 1, 1.33 (1.10–1.63) in Model 2, 1.30 (1.07–1.59) in Model 3, and 1.23 (1.01–1.50) in Model 4.The increasing group was also at a higher risk of non-CVD mortality than was the stable group in all multivariate models. However, no significant difference existed in CVD death in the decreasing group, compared with that of the stable group, across the multivariate models, with adjusted HRs (95% CI) of 1.03 (0.85–1.24) in Model 1, 1.31 (0.84–1.26) in Model 2, 1.02 (0.83–1.24) in Model 3, and 1.02 (0.84–1.25) in Model 4. Figure 5a and b present the forest plots of the results of the subgroup analysis for CVD mortality, considering non-CVD death as a competing risk. The increasing TyG index trajectory group had a significantly higher risk of CVD death in the subgroups of age 50–69 years, obesity, DM, and non-DLD (Fig. 5a). Additionally, the risk of non-CVD death was significantly higher in the increasing TyG index trajectory group than in the stable group for the subgroups of age < 50 years, men, HTN, DM, and DLD (Fig. 5b).

**Table 3:** Competing risk analysis for CVD mortality by trajectory groups of TyG index changes

	TyG index trajectory groups				
	Increasing (n = 64,415)		Stable (n = 112,493)	Decreasing (n = 56,638)	
	HR (95% CI)	*P* value	HR	HR (95% CI)	*P* value
CVD mortality					
Model 1	1.20 (0.99–1.46)	0.063	1 (ref)	1.03 (0.85–1.24)	0.8
Model 2	1.33 (1.10–1.63)	0.004	1 (ref)	1.03 (0.84–1.26)	0.78
Model 3	1.30 (1.07–1.59)	0.009	1 (ref)	1.02 (0.83–1.24)	0.87
Model 4	1.23 (1.01–1.50)	0.041	1 (ref)	1.02 (0.84–1.25)	0.810
Non-CVD mortality					
Model 1	1.05 (0.99–1.11)	0.12	1 (ref)	0.95 (0.90–1.01)	0.077
Model 2	1.16 (1.09–1.23)	< 0.001	1 (ref)	0.96 (0.90–1.01)	0.13
Model 3	1.12 (1.06–1.19)	< 0.001	1 (ref)	0.96 (0.91–1.02)	0.23
Model 4	1.08 (1.02–1.14)	0.014	1 (ref)	0.96 (0.91–1.02)	0.22

Model 1: adjusted for TyG index at baseline

Model 2: adjusted for TyG index at baseline, age, sex, and BMI

Model 3: adjusted for variables used in Model 2 plus smoking status, drinking status, and regular exercise

Model 4: adjusted for variables used in Model 3 plus eGFR, HTN, DM, and DLD

*TyG* triglyceride-glucose, *CVD* cardiovascular disease, *BMI* body mass index, *eGFR* estimated glomerular filtration rate, *HTN* hypertension, *DM* diabetes mellitus, *DLD* dyslipidemia, *HR* hazard ratio, *CI* confidence interval

### Sensitivity analysis

Supplementary Table 3 presents the distribution of individuals across trajectory groups, identified by two different modeling approaches: Gaussian finite mixture modeling and group-based trajectory modeling using fixed-effects modeling. All individuals previously classified in the stable group remained in the stable group, based on group-based trajectory modeling with fixed-effects modeling. However, 26.3% of individuals originally in the increasing group were reassigned to the stable group in the new classification, whereas 54.3% of individuals in the decreasing group were reclassified into the stable group. When analyzed by using an alternative modeling approach, the baseline characteristics of the study population yielded results consistent with those of the main analysis (Supplementary Table 4). The risk of all-cause mortality was significantly higher in the increasing group than in the stable group. By contrast, the all-cause mortality risk was not significantly different between the decreasing group and the stable group (Supplementary Table 5). The risk of CVD mortality and non-CVD mortality was similarly significantly higher in the increasing group than in the stable group (Supplementary Table 6).

After excluding individuals who died within the first 2 years of the event accrual period, the risk of all-cause mortality remained significantly higher in the increasing group than in the stable group (Supplementary Table 7). A significant association between the increasing group and a higher risk of both CVD mortality and non-CVD mortality, compared with the stable group, was evident in Model 2 and Model 3. However, the significance was attenuated in Model 4 (Supplementary Table 8).

## Discussion

Our study unveiled the potential of TyG index changes as a predictive tool for mortality. We found that the increasing TyG index changes group had a higher risk of all-cause and CVD-specific mortality than did the stable group, whereas the risk of CVD death was similar between the decreasing group and stable group.

The influence of the TyG index on adverse health outcomes has garnered attention as a valuable tool for assessing IR. Some studies suggest that the TyG index is linked to adverse all-cause and CVD outcomes in the general population, as well as in high-risk patients, whereas other studies report contradictory results [18–25]. For example, a study of 3,524,459 Chinese adults indicated a reverse L-shaped association between the TyG index and CVD mortality [23], but a meta-analysis demonstrated no association between the TyG index and mortality [25]. These discrepancies highlight the need for further research and are likely caused by limitations such as small sample sizes, short follow-up periods, and differences in population demographics and locations.

Our study utilized a large cohort with an extended follow-up period, allowing for a more robust analysis of how TyG index changes influence mortality. By categorizing participants, based on increasing, stable, or decreasing TyG index trajectories, we better understood the dynamic nature of IR and its relationship with mortality risk. This approach addresses the shortcomings of previous studies that relied solely on baseline IR data and single-point measurements [26, 27]. IR, reflected in the components of the TyG index (i.e., glucose and triglycerides), tends to increase with age because of changes in body fat, insulin signaling, and lipid metabolism [28]; therefore, monitoring the TyG index over time is critical. Glucose variability and rising plasma triglycerides, also associated with CVD risk, further underscore the need to consider these temporal changes [29, 30].

Recent studies have demonstrated the value of tracking TyG index changes over time. For instance, in patients with type 2 DM, the baseline TyG index and its trajectories were both associated with major adverse cardiovascular events [31]. Xu et al. [32] similarly identified three distinct TyG trajectories (i.e., low, moderate, and high) in a younger population, which offered valuable insights into cardiovascular risk. Building on these findings, our study included a broader population across various age groups, making the results more generalizable. By examining these dynamic changes in the TyG index, we better understood how these fluctuations affect long-term cardiovascular and all-cause mortality, thereby enhancing risk stratification and intervention strategies.

Our subgroup analysis revealed higher all-cause mortality in individuals younger than 50, men, and individuals with obesity, HTN, DM, or DLD in the increasing TyG group. Additionally, this group showed higher CVD mortality in individuals aged 50–69 years, individuals with obesity and DM, and individuals without DLD. These findings underscore the importance of monitoring TyG index changes, especially in high-risk populations. In particular, some types of antidiabetic medications such as biguanides and thiazolidinediones can influence serum glucose and insulin levels [33]; therefore, serial monitoring of TyG index changes may be more reliable than monitoring HOMA-IR changes in patients with DM. Further research is needed to investigate the underlying causal mechanisms. Our results align with those of the Global Burden of Disease Study 2019, and highlight the rising CVD burden in the young population [34]. The increasing prevalence of DM and obesity in adults younger than 45 years contributes to this trend. Previous studies also demonstrate that persistently elevated TyG index levels in young adulthood are associated with increased CVD and mortality risks later in life [32, 35], which reinforces our findings.

The mechanism linking TyG index changes with increased mortality remains unclear, but several possible explanations have been proposed. First, higher insulin and insulin-like growth factor I levels may be secreted in response to the gradual rise in IR, leading to cellular proliferation and reduced apoptosis, which could contribute to increased carcinogenicity [36]. Second, the gradual time-dependent increase in the TyG index may lead to a greater accumulation of oxidative stress due to hypertriglyceridemia, thereby causing endothelial dysfunction and contributing to the initiation and progression of atherosclerosis [37]. Finally, prolonged exposure to hyperglycemia may promote the glycation of platelet proteins, enhance platelet reactivity and potentially increase the risk of CVD death [38].

To our knowledge, this study is the first to explore the association between long-term TyG index trajectories and all-cause and CVD mortality in a large national cohort. By leveraging a robust sample size and linking mortality data with official records from the Korea National Statistical Office, we captured dynamic changes in IR over time, providing a more comprehensive understanding of its impact on mortality risk.

Several limitations should be noted in this study. First, although we adjusted for well-known confounders, a causal relationship between TyG index trajectories and mortality could not be confirmed. Future research should consider large-scale randomized controlled trials or Mendelian randomization studies to establish causality.

Second, although TyG index changes were modeled as a time-dependent variable, other factors that also fluctuate over time such as smoking habit, alcohol consumption, and exercise were not included as time-dependent covariates. Owing to the small proportion of changes in these lifestyle factors and modeling complexity, we used only baseline values. Additionally, missing data prevented us from adjusting for socioeconomic status, potentially allowing confounding effects [39], despite adjusting for well-known risk factors for CVD mortality [35, 40, 41]. Third, our cohort likely reflected a healthier population because the participants completed three consecutive health screenings. The lower CVD mortality rate may also be partially explained by the more specific definition of CVD used in our study than that used by the 2009–2010 Korea National Health Screening Program cohort [42]. Furthermore, because this study included only Korean participants, external validation in other ethnicities is necessary to generalize the findings.

## Conclusion

For individuals with a set TyG index at baseline, an increase in TyG index levels during follow-up was independently associated with a higher risk of all-cause and CVD mortality. These findings underscore the importance of serial monitoring of TyG index changes and prioritizing strategies to reduce IR to lower mortality risk.

## Supplementary Information

Supplementary Material 1.

## Data Availability

The data analyzed in this study cannot be shared by the authors because the data are owned by the Korean National Health Insurance Service (NHIS). Researchers can apply for access to the data via the NHIS website (https://nhiss.nhis.or.kr). Comprehensive details on the process and a provision guide are available at http://nhiss.nhis.or.kr/bd/ab/bdaba000eng.do. No datasets were generated or analysed during the current study.

## Abbreviations

CVD: Cardiovascular disease
IR: Insulin resistance
HOMA-IR: Homeostatic model assessment for insulin resistance
TyG: Triglyceride-glucose
NHIS-NSC: National Health Insurance Service-National Sample Cohort
ICD-10: International Classification of Diseases, tenth revision
FBG: Fasting blood glucose
BMI: Body mass index
SBP: Systolic blood pressure
DBP: Diastolic blood pressure
HDL-C: High-density lipoprotein cholesterol
eGFR: Estimated glomerular filtration rate
DM: Diabetes mellitus
HTN: Hypertension
DLD: Dyslipidemia
HR: Hazard ratio
CI: Confidence interval
