# Supplementary material for: Trajectories of triglyceride-glucose index changes and their association with all-cause and cardiovascular mortality: a competing risk analysis
Source: Cardiovasc Diabetol. 2024 Oct 15;23:364. doi: 10.1186/s12933-024-02457-y (PMC11481394; doi:10.1186/s12933-024-02457-y)
Supplement: Supplementary file 1 — Supplementary Material 1. [file 12933_2024_2457_MOESM1_ESM.docx]

**Supplementary Table 1.** Estimation process for the most optimal number of TyG index trajectories.

|  |  |  |  |  |  | **Average posterior probability assignment** | | | |
| --- | --- | --- | --- | --- | --- | --- | --- | --- | --- |
| **Number of groups** | **Group 1** | **Group 2** | **Group 3** | **Group 4** | **BIC** | **Group 1** | **Group 2** | **Group 3** | **Group 4** |
| 2 | 108,832 (47%) | 124,714 (53%) |  |  | 736076.5 | 90.0% | 89.4% |  |  |
| 3 | 64,415 (28.9%) | 56,638 (25.7%) | 112,493 (45.4%) |  | 628333.4 | 90.0% | 89.9% | 84.0% |  |
| 4 | 31030 (15.1%) | 79,975 (32.5%) | 42,806 (20.2%) | 79,735 (32.1%) | 580197.2 | 80.3% | 87.7% | 82.0% | 87.7% |

Abbreviations: TyG, triglyceride-glucose; BIC, Bayesian information criterion.

**Supplementary Table 2.** Subgroup analysis for the risk of all-cause mortality

|  |  | **TyG index trajectory groups** | | | | |
| --- | --- | --- | --- | --- | --- | --- |
|  |  | **Increasing** |  | **Stable** | **Decreasing** | |
| **All-cause mortality^*^** | **Number** | **HR (95% CI)** | ***P* value** | **HR** | **HR (95% CI)** | ***P* value** |
| **Age subgroup** |  |  |  |  |  |  |
| <50 years | 128,833 | 1.22 (1.03–1.44) | 0.02 | 1 (ref) | 0.99 (0.82–1.19) | 0.927 |
| 50–69 years | 90,990 | 0.98 (0.91–1.05) | 0.71 | 1 (ref) | 0.97 (0.90–1.05) | 0.493 |
| ≥70 years | 13,723 | 1.08 (0.99–1.19) | 0.073 | 1 (ref) | 0.98 (0.89–1.07) | 0.626 |
| **Sex subgroup** |  |  |  |  |  |  |
| Men | 125,249 | 1.09 (1.02–1.17) | 0.012 | 1 (ref) | 0.97 (0.90–1.04) | 0.338 |
| Women | 108,297 | 1.07 (0.97–1.18) | 0.19 | 1 (ref) | 0.99 (0.89–1.09) | 0.807 |
| **Obesity subgroup** |  |  |  |  |  |  |
| Obese | 78,004 | 1.14 (1.02–1.26) | 0.015 | 1 (ref) | 0.93 (0.84–1.03) | 0.182 |
| Normal weight | 155,447 | 1.06 (0.99–1.13) | 0.113 | 1 (ref) | 1.00 (0.94–1.08) | 0.912 |
| **HTN subgroup** |  |  |  |  |  |  |
| HTN | 90,003 | 1.09 (1.02–1.17) | 0.01 | 1 (ref) | 0.99 (0.93–1.06) | 0.832 |
| Non-HTN | 143,523 | 1.08 (0.97–1.20) | 0.15 | 1 (ref) | 0.91 (0.81–1.01) | 0.085 |
| **DM subgroup** |  |  |  |  |  |  |
| DM | 19,268 | 1.33 (1.17–1.52) | <0.001 | 1 (ref) | 0.95 (0.84–1.08) | 0.421 |
| Non-DM | 214,278 | 1.04 (0.97–1.10) | 0.278 | 1 (ref) | 0.98 (0.92–1.05) | 0.546 |
| **DLD subgroup** |  |  |  |  |  |  |
| DLD | 31,490 | 1.27 (1.09–1.49) | 0.002 | 1 (ref) | 0.89 (0.77–1.04) | 0.14 |
| Non-DLD | 202,056 | 1.46 (1.37–1.56) | 0.056 | 1 (ref) | 0.98 (0.92–1.04) | 0.552 |

^*^adjusted for TyG index at baseline, age, sex, BMI, smoking status, drinking status, regular exercise, eGFR, HTN, DM, and DLD.

Variables corresponding to each subgroup were excluded from the models.

Abbreviations: TyG, triglyceride-glucose; BMI, body mass index; eGFR, estimated glomerular filtration rate; HTN, hypertension, DM, diabetes mellitus; DLD, dyslipidemia; HR, hazard ratio, CI, confidence interval.

**Supplementary Table 3.** Sensitivity analysis 1: Comparisson of trajectory group matching for TyG index changes using Gaussian finite mixture modeling and group-based trajectory modeling through fixed-effects modeling

|  | | Gaussian finite mixture modeling | | |  |
| --- | --- | --- | --- | --- | --- |
|  |  | Increasing | Stable | Decreasing |  |
| Group-based trajectory modeling through fixed-effects modeling | Increasing | 47,470 |  | 1 | 47,471 |
|  | Stable | 16,945 | 112,493 | 30,223 | 155,852 |
|  | Decreasing |  |  | 26,414 | 30,223 |
|  | | 64,415 | 112,493 | 55,638 |  |

Abbreviation: TyG, triglyceride-glucose.

**Supplementary Table 4.** Sensitivity analysis 1: Clinical characteristics of the study population based on the TyG index trajectories using group-based trajectory modeling through fixed-effects modeling

|  | TyG index trajectory groups | | |  |
| --- | --- | --- | --- | --- |
|  | Increasing | Stable | Decreasing | *P* value |
|  | (n = 47,471) | (n = 155,852) | (n = 30,223) |  |
| Men, n (%) | 26,227 (55.2%) | 81,748 (52.5%) | 17,274 (57.2%) | <0.001 |
| Age, years | 46.3 ± 13.3 | 48.0 ± 13.3 | 49.7 ± 13.1 | <0.001 |
| BMI, kg/m^2^ | 23.7 ± 3.1 | 23.7 ± 3.1 | 24.2 ± 3.1 | <0.001 |
| SBP, mmHg | 121.5 ± 14.5 | 122.2 ± 14.8 | 124.8± 15.1 | <0.001 |
| DBP, mmHg | 75.7 ± 9.9 | 76.2 ± 9.9 | 77.7 ± 10.0 | <0.001 |
| FBG, mg/dL | 95.5 ± 17.4 | 96.4 ± 18.99 | 108.7 ± 37.8 | <0.001 |
| Total cholesterol, mg/dL | 191.1 ± 38.5 | 195.9 ± 40.0 | 203.7 ± 46.1 | <0.001 |
| Triglyceride, mg/dL | 78 (56, 113) | 110 (78, 157) | 183 (128, 268) | <0.001 |
| HDL-C, mg/dL | 55 (46, 64) | 53 (45, 63) | 51 (43, 60) | <0.001 |
| Creatinine, mg/dL | 0.9 (0.8, 1.0) | 0.9 (0.8, 1.0) | 0.9 (0.8, 1.1) | <0.001 |
| eGFR, mL/min/1.73m^2^ | 80.1 (70.3, 92.1) | 79.1 (69.3, 90.9) | 78.2 (68.1, 90.1) | <0.001 |
| Current smoker, n (%) | 12,536 (26.5%) | 34,837 (22.5%) | 6986 (23.2%) | <0.001 |
| Current drinker, n (%) | 23,062 (49.4%) | 71,044 (46.3%) | 14,203 (47.8%) | <0.001 |
| Regular exerciser, n (%) | 12,568 (26.8%) | 39,097 (25.3%) | 7527 (25.2%) | <0.001 |
| HTN, n (%) | 17,111 (36.0%) | 58,992 (37.9%) | 13,900 (46.0%) | <0.001 |
| DM, n (%) | 3248 (6.8%) | 10,945 (7.0%) | 5075 (16.8%) | <0.001 |
| DLD, n (%) | 5319 (11.2%) | 20,619 (13.2%) | 5552 (18.4%) | <0.001 |
| TyG index |  |  |  |  |
| 2009–2010 | 8.2 ± 0.6 | 8.6 ± 0.6 | 9.2 ± 0.6 | <0.001 |
| 2011–2012 | 8.8 ± 0.7 | 8.6 ± 0.6 | 8.5 ± 0.7 | <0.001 |
| 2013–2014 | 9.0 ± 0.7 | 8.6 ± 0.6 | 8.3 ± 0.6 | <0.001 |

Abbreviations: TyG, triglyceride-glucose; BMI, body mass index; SBP, systolic blood pressure; DBP, diastolic blood pressure; FBG, fasting blood glucose; HDL-C, high-density lipoprotein cholesterol; eGFR, estimated glomerular infiltration rate; HTN, hypertension; DM, diabetes mellitus; DLD, dyslipidemia.

**Supplementary Table 5.** Sensitivity analysis 1: Cox proportional hazard regression analysis for all-cause mortality by trajectory groups of TyG index changes using group-based trajectory modeling through fixed-effects modeling

|  | TyG index trajectory groups | | | | |
| --- | --- | --- | --- | --- | --- |
|  | Increasing  (n = 47,471) |  | Stable  (n = 155,852) | Decreasing  (n = 30,223) | |
| All-cause moratlity | HR (95% CI) | *P* value | HR | HR (95% CI) | *P* value |
| Model 1 | 1.09 (1.02–1.15) | 0.007 | 1 (ref) | 0.95 (0.89–1.02) | 0.152 |
| Model 2 | 1.21 (1.14–1.29) | <0.001 | 1 (ref) | 0.95 (0.89–1.01) | 0.118 |
| Model 3 | 1.18 (1.11–1.25) | <0.001 | 1 (ref) | 0.96 (0.89–1.02) | 0.203 |
| Model 4 | 1.12 (1.05–1.19) | <0.001 | 1 (ref) | 0.96 (0.90–1.03) | 0.234 |

Model 1: adjusted for TyG index at baseline.

Model 2: adjusted for TyG index at baseline, age, sex, and BMI.

Model 3: adjusted for variables used in Model 2 plus smoking status, drinking status, and regular exercise.

Model 4: adjusted for variables used in Model 3 plus eGFR, HTN, DM, and DLD.

Abbreviations: TyG, triglyceride-glucose; BMI, body mass index; eGFR, estimated glomerular filtration rate; HTN, hypertension; DM, diabetes mellitus; DLD, dyslipidemia; HR, hazard ratio; CI, confidence interval.

**Supplementary Table 6.** Sensitivity analysis 1: Competing risk analysis for CVD mortality by trajectory groups of TyG index changes using group-based trajectory modeling through fixed-effects modeling

|  | TyG index trajectory groups | | | | |
| --- | --- | --- | --- | --- | --- |
|  | Increasing  (n = 64,415) |  | Stable  (n = 112,493) | Decreasing  (n = 56,638) | |
| CVD mortality | HR (95% CI) | *P* value | HR | HR (95% CI) | *P* value |
| Model 1 | 1.33 (1.09–1.63) | 0.006 | 1 (ref) | 1.00 (0.80–1.25) | 0.98 |
| Model 2 | 1.50 (1.21–1.84) | <0.001 | 1 (ref) | 0.98 (0.78–1.24) | 0.89 |
| Model 3 | 1.48 (1.20–1.82) | <0.001 | 1 (ref) | 0.97 (0.77–1.23) | 0.8 |
| Model 4 | 1.39 (1.12–1.71) | 0.003 | 1 (ref) | 0.98 (0.78–1.24) | 0.87 |
| Non-CVD mortality | HR (95% CI) | *P* value | HR | HR (95% CI) | *P* value |
| Model 1 | 1.06 (1.00–1.13) | 0.049 | 1 (ref) | 0.95 (0.89–1.01) | 0.13 |
| Model 2 | 1.18 (1.11–1.26) | <0.001 | 1 (ref) | 0.95 (0.88–1.01) | 0.12 |
| Model 3 | 1.15 (1.08–1.22) | <0.001 | 1 (ref) | 0.96 (0.89–1.03) | 0.22 |
| Model 4 | 1.10 (1.03–1.17) | 0.005 | 1 (ref) | 0.96 (0.89–1.03) | 0.24 |

Model 1: adjusted for TyG index at baseline.

Model 2: adjusted for TyG index at baseline, age, sex, and BMI.

Model 3: adjusted for variables used in Model 2 plus smoking status, drinking status, and regular exercise.

Model 4: adjusted for variables used in Model 3 plus eGFR, HTN, DM, and DLD.

Abbreviations: TyG, triglyceride-glucose; CVD, cardiovascular disease; BMI, body mass index; eGFR, estimated glomerular filtration rate; HTN, hypertension; DM, diabetes mellitus; DLD, dyslipidemia; HR, hazard ratio; CI, confidence interval.

**Supplementary Table 7.** Sensitivity analysis 2: Cox proportional hazard regression analysis for all-cause mortality by trajectory groups of TyG index changes after excluding individuals who died within the first 2 years

|  | TyG index trajectory groups | | | | |
| --- | --- | --- | --- | --- | --- |
|  | Increasing  (n = 66,687) |  | Stable  (n = 110,844) | Decreasing  (n = 55,250) | |
| All-cause mortality | HR (95% CI) | *P* value | HR | HR (95% CI) | *P* value |
| Model 1 | 1.04 (0.98–1.10) | 0.225 | 1 (ref) | 0.96 (0.90–1.02) | 0.187 |
| Model 2 | 1.14 (1.08–1.21) | <0.001 | 1 (ref) | 0.97 (0.91–1.03) | 0.317 |
| Model 3 | 1.11 (1.05–1.18) | <0.001 | 1 (ref) | 0.97 (0.92–1.04) | 0.411 |
| Model 4 | 1.07 (1.01–1.13) | 0.031 | 1 (ref) | 0.97 (0.92–1.04) | 0.394 |

Model 1: adjusted for TyG index at baseline.

Model 2: adjusted for TyG index at baseline, age, sex, and BMI.

Model 3: adjusted for variables used in Model 2 plus smoking status, drinking status, and regular exercise.

Model 4: adjusted for variables used in Model 3 plus eGFR, HTN, DM, and DLD.

Abbreviations: TyG, triglyceride-glucose; CVD, cardiovascular disease; BMI, body mass index; eGFR, estimated glomerular filtration rate; HTN, hypertension; DM, diabetes mellitus; DLD, dyslipidemia; HR, hazard ratio; CI, confidence interval.

**Supplementary Table 8.** Sensitivity analysis 2: Competing risk analysis for CVD mortality by trajectory groups of TyG index changes after excluding individuals who died within the first 2 years

|  | TyG index trajectory groups | | | | |
| --- | --- | --- | --- | --- | --- |
|  | Increasing  (n = 66,687) |  | Stable  (n = 110,844) | Decreasing  (n = 55,250) | |
| CVD death | HR (95% CI) | *P* value | HR | HR (95% CI) | *P* value |
| Model 1 | 1.21 (0.98–1.49) | 0.077 | 1 (ref) | 1.04 (0.84–1.29) | 0.69 |
| Model 2 | 1.33 (1.08–1.65) | 0.008 | 1 (ref) | 1.06 (0.85–1.32) | 0.61 |
| Model 3 | 1.30 (1.05–1.61) | 0.015 | 1 (ref) | 1.04 (0.83–1.29) | 0.74 |
| Model 4 | 1.24 (1.00–1.53) | 0.053 | 1 (ref) | 1.04 (0.84–1.30) | 0.71 |
| Non-CVD death | HR (95% CI) | *P* value | HR | HR (95% CI) | *P* value |
| Model 1 | 1.02 (0.96–1.09) | 0.46 | 1 (ref) | 0.95 (0.90–1.01) | 0.13 |
| Model 2 | 1.13 (1.06–1.20) | <0.001 | 1 (ref) | 0.96 (0.90–1.03) | 0.23 |
| Model 3 | 1.10 (1.03–1.17) | 0.003 | 1 (ref) | 0.97 (0.91–1.03) | 0.34 |
| Model 4 | 1.06 (0.99–1.12) | 0.085 | 1 (ref) | 0.97 (0.91–1.03) | 0.31 |

Model 1: adjusted for TyG index at baseline.

Model 2: adjusted for TyG index at baseline, age, sex, and BMI.

Model 3: adjusted for variables used in Model 2 plus smoking status, drinking status, and regular exercise.

Model 4: adjusted for variables used in Model 3 plus eGFR, HTN, DM, and DLD.

Abbreviations: TyG, triglyceride-glucose; CVD, cardiovascular disease; BMI, body mass index; eGFR, estimated glomerular filtration rate; HTN, hypertension; DM, diabetes mellitus; DLD, dyslipidemia; HR, hazard ratio; CI, confidence interval.
